# High Expression Levels of BLyS/BAFF by Blood Dendritic Cells and Granulocytes Are Associated with B-cell dysregulation in SIV-Infected Rhesus Macaques

**DOI:** 10.1371/journal.pone.0131513

**Published:** 2015-06-24

**Authors:** Johanne Poudrier, Caroline Soulas, Josiane Chagnon-Choquet, Tricia Burdo, Patrick Autissier, Kathryn Oskar, Kenneth C. Williams, Michel Roger

**Affiliations:** 1 Laboratoire d’immunogénétique, Centre de Recherche du Centre Hospitalier de l’Université de Montréal (CRCHUM), Montréal, Canada; 2 Département de Microbiologie, Infectiologie et Immunologie de l‘Université de Montréal, Montréal, Canada; 3 Boston College, Boston, MA, United States of America; Emory University School of Medicine, UNITED STATES

## Abstract

Dendritic cells (DCs) modulate B-cell survival and differentiation, mainly through production of growth factors such as B lymphocyte stimulator (BLyS/BAFF). In recent longitudinal studies involving HIV-1-infected individuals with different rates of disease progression, we have shown that DCs were altered in number and phenotype in the context of HIV-1 disease progression and B-cell dysregulations were associated with increased BLyS/BAFF expression in plasma and by blood myeloid DCs (mDCs) in rapid and classic progressors but not in HIV-1-elite controllers (EC). Suggesting that the extent to which HIV-1 disease progression is controlled may be linked to BLyS/BAFF expression status and the capacity to orchestrate B-cell responses. Herein, longitudinal analyses of simian immunodeficiency virus (SIV)-infected rhesus macaques also revealed increased expression of BLyS/BAFF by blood mDCs as soon as day 8 and throughout infection. Strikingly, granulocytes presented the highest BLyS/BAFF expression profile in the blood of SIV-infected macaques. BLyS/BAFF levels were also increased in plasma and correlated with viral loads. Consequently, these SIV-infected animals had plasma hyperglobulinemia and reduced blood B-cell numbers with altered population frequencies. These data underscore that BLyS/BAFF is associated with immune dysregulation in SIV-infected rhesus macaques and suggest that BLyS/BAFF is a key regulator of immune activation that is highly conserved among primates. These findings emphasize the potential importance of this SIV-infected primate model to test whether blocking excess BLyS/BAFF has an effect on the overall inflammatory burden and immune restoration.

## Introduction

Based on the study of natural “immunity/resistance” and on promising vaccine strategies, B-cell responses are now considered to be major players in the battle against HIV-1 [1,2]. Unfortunately, the contribution of the B-cell compartment to effective viral control is impeded in the vast majority of HIV-1-infected individuals. Indeed, B-cell dysregulations including polyclonal activation, breakage of tolerance, altered population dynamics, exhaustion, and the progressive loss of the capacity to generate and maintain memory, are observed early and persist throughout the infection, and are not fully restored by therapy. These alterations impair immune efficiency and favour the overall inflammatory burden and often lead to autoimmune manifestations and malignancies [3,4].

Dendritic cells (DCs) modulate B-cell survival and differentiation, mainly through production of growth factors such as B lymphocyte stimulator (BLyS)/BAFF [5–8]. Early data supporting the role of DCs and BLyS/BAFF in promoting B-cell dysregulation and HIV-1 disease progression were obtained from HIV-transgenic mice, which develop a disease dependent on *nef* and comparable to many aspects of human AIDS [9,10]. These animals had an enlarged splenic marginal zone (MZ), in which accumulation of myeloid DCs (mDCs) likely contributed to MZ expansion, polyclonal B-cell activation and breakage of tolerance through delivery of excessive signals such as BLyS/BAFF [11,12]. Accordingly, BLyS/BAFF, of which DCs are significant and primary producers, highly influence the transitional immature (TI) and MZ B-cell pools [13,14]. A similar B-cell profile was reported for BLyS/BAFF-transgenic [15] and autoimmune-regulatory-(AIRE)-deficient mice, in which BLyS/BAFF is elevated in serum and over-expressed by DCs [16,17].

In recent longitudinal studies of HIV-1-infected individuals with different rates of disease progression, we have shown that DCs were altered in number and phenotype in HIV-1 progressors when compared to HIV-1-elite controllers and HIV-negative donors [18]. Moreover, high levels of BLyS/BAFF in plasma and on the surface of blood mDCs were observed in HIV-1 progressors, as soon as in the acute phase and persisted throughout infection despite highly active therapy [19]. Accordingly, these individuals presented B-cell dysregulations reflected by serum hyperglobulinemia and reduced percentages of total blood B-cells when compared to HIV-negative donors. However, the relative frequencies of a population presenting features shared by both TI and recirculating first-line MZ B-cells, designated as “precursor” MZ-like B-cells, were increased in the blood of HIV-1 progressors [19,20].

We aimed to longitudinally monitor cell populations and BLyS/BAFF expression in the blood of SIV-infected rhesus macaques to determine whether BLyS/BAFF influences SIV-disease progression similarly to that reported in HIV-infected humans. Additionally, we sought to determine the utility of SIV-infected rhesus monkeys for future studies targeting BLyS/BAFF.

## Materials and Methods

### Ethical Statement

All animals used in this study were handled in strict accordance with American Association for Accreditation of Laboratory Animal Care with the approval of the Institutional Animal Care and Use Committee of Harvard University. The New England Primate Research Center (NEPRC) Protocol Number for this study is 04420 and the Animal Welfare Assurance Number is A3431-01. After infection with SIV, animals were individually housed, but received all other components of the NEPRC Environmental Enrichment Program. The enrichment program was supervised by NEPRC veterinarians in collaboration with Animal Behavioral staff, not by the PI. Enrichment was provided through manipulatable devices, food items, structural and environmental enhancements, and positive human interaction. Animals did not undergo food or water deprivation at any time during the study and were monitored daily for evidence of disease and changes in appetite and behavior. All possible measures are taken to minimize discomfort of the animals. All procedures were performed using chemical restraint to ensure the safety of both staff and animals and the choice of anesthetic includes ketamine (10–20 mg/kg, IM), telazol (4–10 mg/kg, IM), and/or dexdomitor (7.5–15 μg/kg, IM), depending on the procedure.

### Animals

Five male rhesus macaques, age 4–6 yrs, were infected with SIVmac251 (2 ng of SIV p27) by intravenous injection. Sivmac251 was kindly provided by Dr Ronald Desrosiers (formerly at Harvard University’s New England Regional Primate Research Center, now director of Miller School’s Department of Pathology at University of Miami Health System). Animals were followed for a period of 1–2 yrs, and blood samples were collected at the following time points: day -7 and day 0 prior to infection, in the acute phase of infection at days 8 and 14, in the asymptomatic phase of infection at days 56–76, in the chronic phase at day 182 post-infection, and finally at necropsy. Fresh blood samples were used for FACS analysis of leukocytes immediately following venous puncture [21] and plasma were frozen at –20°C. Plasma SIV viral loads were determined by qRT-PCR as described [22]. The threshold sensitivity was 30 copy Equivalents SIV RNA per ml for a typical input of 0.5 ml plasma. Animals were anesthetized with ketamine-HCL and euthanized by an intravenous pentobarbital overdose and exsanguinated when presenting any of the following 11 criteria: 1) weight loss >15% body weight in 2 weeks or >25% body weight overall, 2) documented opportunistic infection, 3) persistent anorexia >3 days without explicable cause, 4) severe intractable diarrhea that is none responsive to standard treatment and results in dehydration and debilitation of the animal, 5) progressive neurological signs, 6) significant cardiac and/or pulmonary signs, 7) persistent leucopenia or thrombocytopenia, 8) progressive or persistent anemia, 9) CD4 depletion or other signs of progressive immunosuppressive disease, 10) body condition score <1.5/5 with weight loss or 11) any other serious illness. Animals # 32, 33, 34, 35, and 36 were euthanized at days 461, 210, 552, 266, and 400 post-infection, respectively.

### Evaluation of blood cell populations and BLyS/BAFF expression by flow-cytometry

Analysis by 12-panel multi-color flow-cytometry on fresh blood samples was performed according to the previously published protocol [21]. Cells were labeled with the following cocktail of anti-human monoclonal antibodies: FITC-conjugated Anti-IgM, PE-conjugated Anti-CD27, PE-Cy5-conjugated Anti-CD21, PerCP-Cy5.5-conjugated Anti-CD16, PE-Cy7 Anti-CD10, Alexa Fluor 700-conjugated Anti-CD3, Qdot 605-conjugated Anti-CD4, APC-Cy7-conjugated Anti-CD20, Pacific Blue-conjugated Anti-CD14 (Becton Dickinson (BD) Biosciences, San Jose, CA, USA); ECD-conjugated Anti-HLA-DR (Beckman Coulter); APC-conjugated Anti-CD1c (Miltenyi Biotec); (Qdot 655) biotinylated Anti-human CD257 (BLyS/BAFF) (eBioscience, San Diego, CA, USA). Data acquisition of 100,000 events per sample was performed using a FACSAria (BD-Biosciences) Flow-Cytometer, and analysis was done with FlowJo7.6.3 software (TreeStar, Ashland, OR, USA). All staining were compared to that of fluorescence minus one (FMO) values and isotype controls. Anti-mouse Ig(κ) CompBeads and CS&T Beads (BD-Biosciences) were used to optimize fluorescence compensation settings and calibrate the FACSAria, respectively.

### Evaluation of BLyS/BAFF and immunoglobulin concentrations

Plasma levels of BLyS/BAFF and immunoglobulin (Ig) were measured using commercially available anti-human BLyS/BAFF ELISA kit (R&D Systems, Minneapolis, MN, USA), and anti-human IgM and IgG ELISA kits (Life Diagnostics, West Chester PA, USA) respectively, and performed according to the manufacturer’s protocols.

### Statistical analyses

Parameters at time points before infection (controls) were compared to those after infection using paired Student T-tests. Repeated-measures ANOVA followed by Tukey’s post-test were used for pairwise comparison between time points. Correlations between levels of plasma viral loads and BLyS/BAFF were done by Pearson test. Analyses were performed using GraphPad Prism 5.03 for Windows (GraphPad Software Inc, La Jolla, CA, USA).

## Results

### Plasma viral loads during SIV infection in macaques

**Fig 1:**
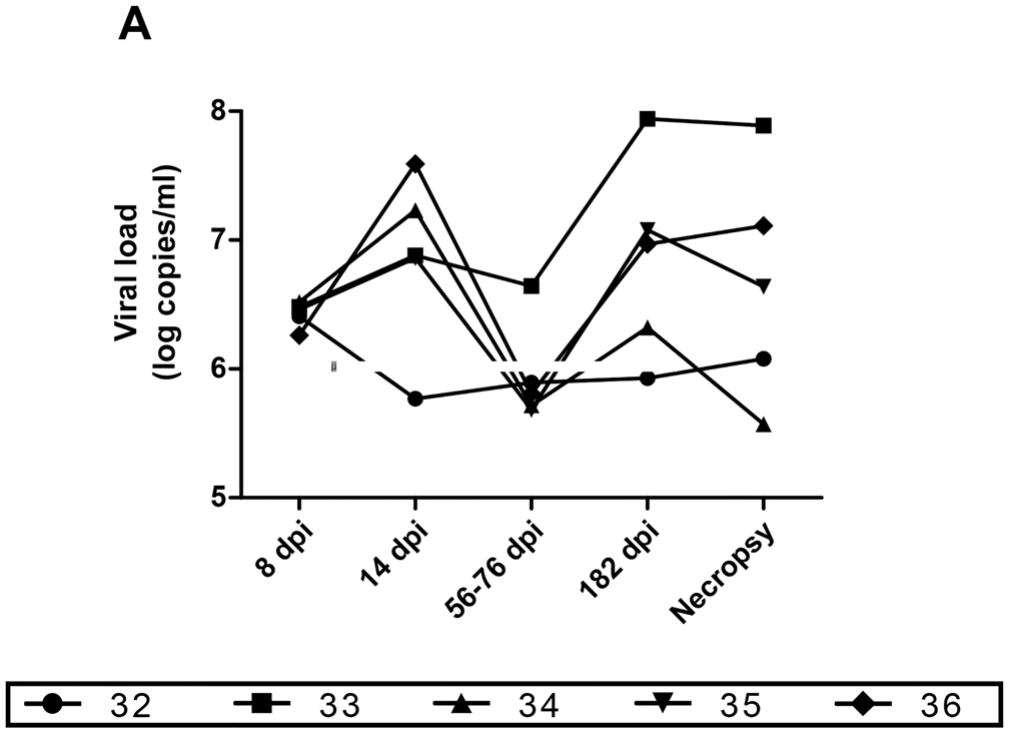
Longitudinal monitoring of plasma SIV viral loads. Viral loads (log copies/ml) of 5 SIV-infected macaques were quantified by quantitative RT-PCR and compared to values obtained at pre-infection stage using the paired Student's t test. No significant differences were observed between animals. dpi, days post-infection.

Plasma virus was initially assessed at day 8 post-infection and levels of viral RNA fluctuated throughout infection with relatively high levels during the acute (day 14) and chronic (day 182 and necropsy) infection, and low levels at days 56–76 post-infection (Fig 1). One animal, #32 had a relatively low level of viremia that remained stable after 14 days of infection and onwards. Whereas animal #34 presented a viral load that tended to be low following the acute phase.

### Longitudinal monitoring of granulocytes, CD4^+^ T-cells and myeloid cell populations in the blood of SIV-infected macaques

**Fig 2:**
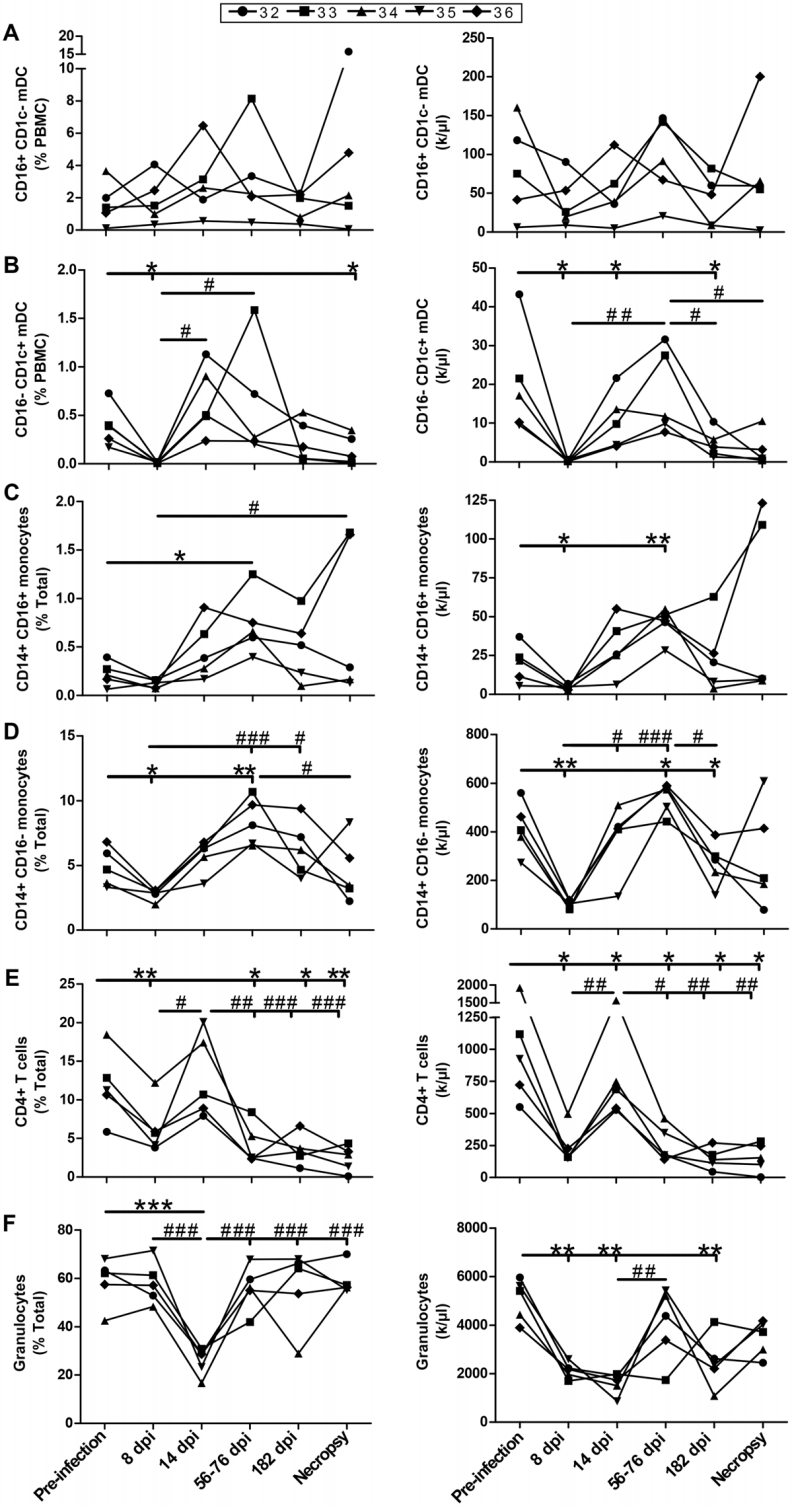
Longitudinal flow-cytometry analysis of blood myeloid dendritic cells (mDC), monocytes, CD4^+^ T-cells and granulocytes. Relative percentages (left panels) and absolute numbers (right panels) of (A) CD16^+^CD1c^-^ mDC, (B) CD16^-^CD1c^+^ mDC, (C) CD14^+^CD16^+^ monocytes, (D) CD14^+^CD16^-^ monocytes, (E) CD4^+^ T-cells and (F) granulocytes obtained from the blood of 5 SIV-infected rhesus macaques. Values were compared to that obtained at pre-infection using the paired Student's *t* test (*) or between time points with repeated-measures ANOVA test followed by Tukey’s post-test (#). Significances levels are shown as *,# p < 0.05, **,## p < 0.001 and ***,###. dpi, days post-infection.

A representative scheme showing the flow-cytometry gating strategy used for the analysis of macaque blood granulocytes, monocytes, CD4^+^ T-cells and mDCs is provided in S1 Fig. Both frequencies and absolute numbers of mDC, monocyte and granulocyte populations in the blood of SIV-infected macaques were decreased in the acute (day 8) and chronic (day 182 and necropsy) phases of infection, with restoration at days 56–76 post-infection (Fig 2). Both the frequencies and absolute numbers of CD4^+^ T-cells decreased steadily throughout infection albeit we observed a tentative of restoration at day 14 post-infection (Fig 2E).

### Longitudinal monitoring of BLyS/BAFF expression levels in the blood of SIV-infected macaques

**Fig 3:**
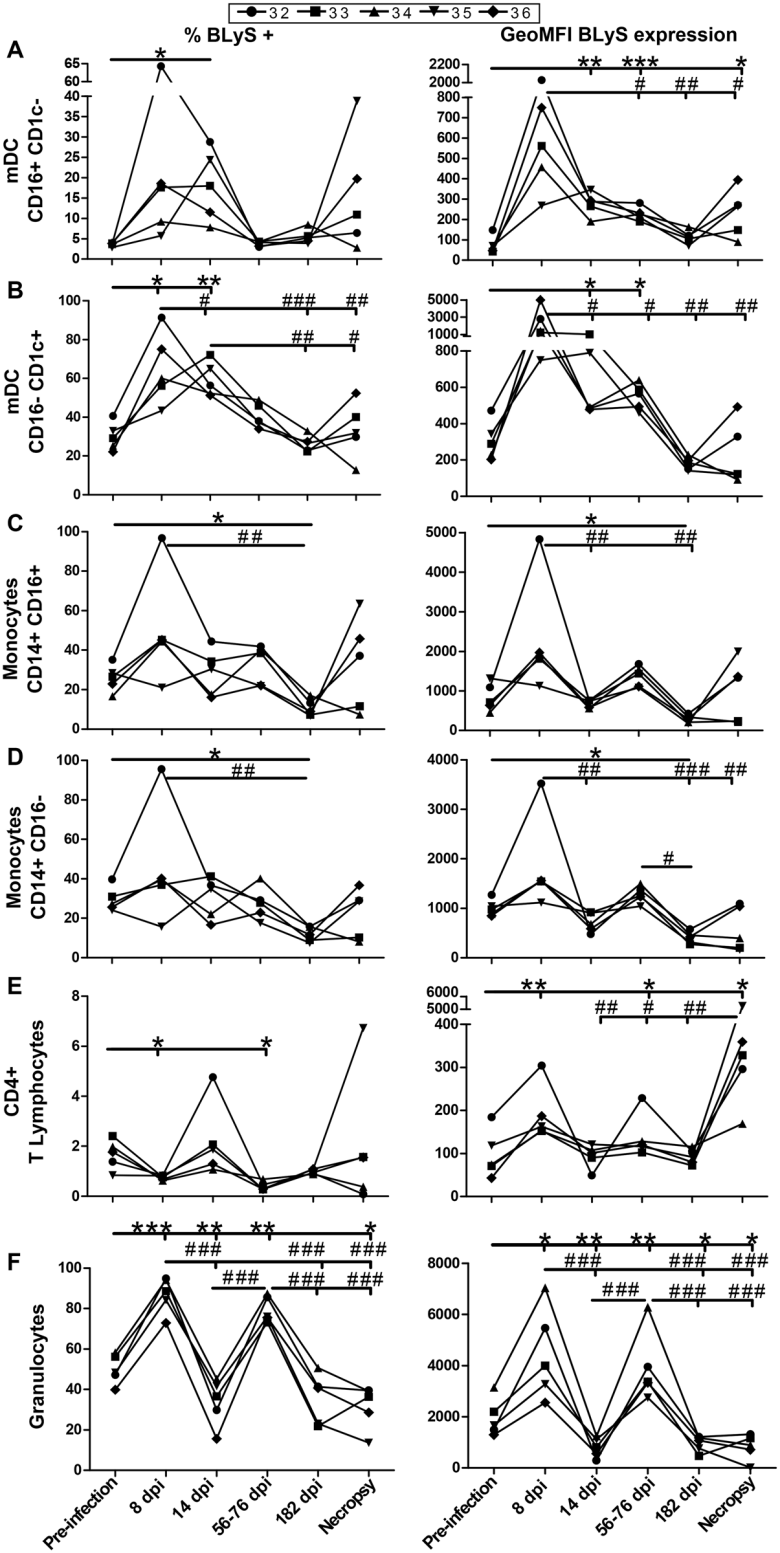
Longitudinal flow-cytometry analysis of BLyS/BAFF expression by blood myeloid dendritic cells (mDC), monocytes, CD4^+^ T-cells and granulocytes. Relative percentages (left panels) and geometric mean fluorescence intensity (GeoMFI) (right panels) of BLyS/BAFF expression on (A) CD16^+^CD1c^-^ mDC, (B) CD16^-^CD1c^+^ mDC, (C) CD14^+^CD16^+^ monocytes, (D) CD14^+^CD16^-^ monocytes, (E) CD4^+^ T-cells and (F) granulocytes obtained from the blood of 5 SIV-infected rhesus macaques. Values were compared to that obtained at pre-infection using the paired Student's *t* test (*) or between time points with repeated-measures ANOVA test followed by Tukey’s post-test (#). Significances levels are shown as *,# p < 0.05, **,## p < 0.001 and ***,###p < 0.0001. dpi, days post-infection.

**Fig 4:**
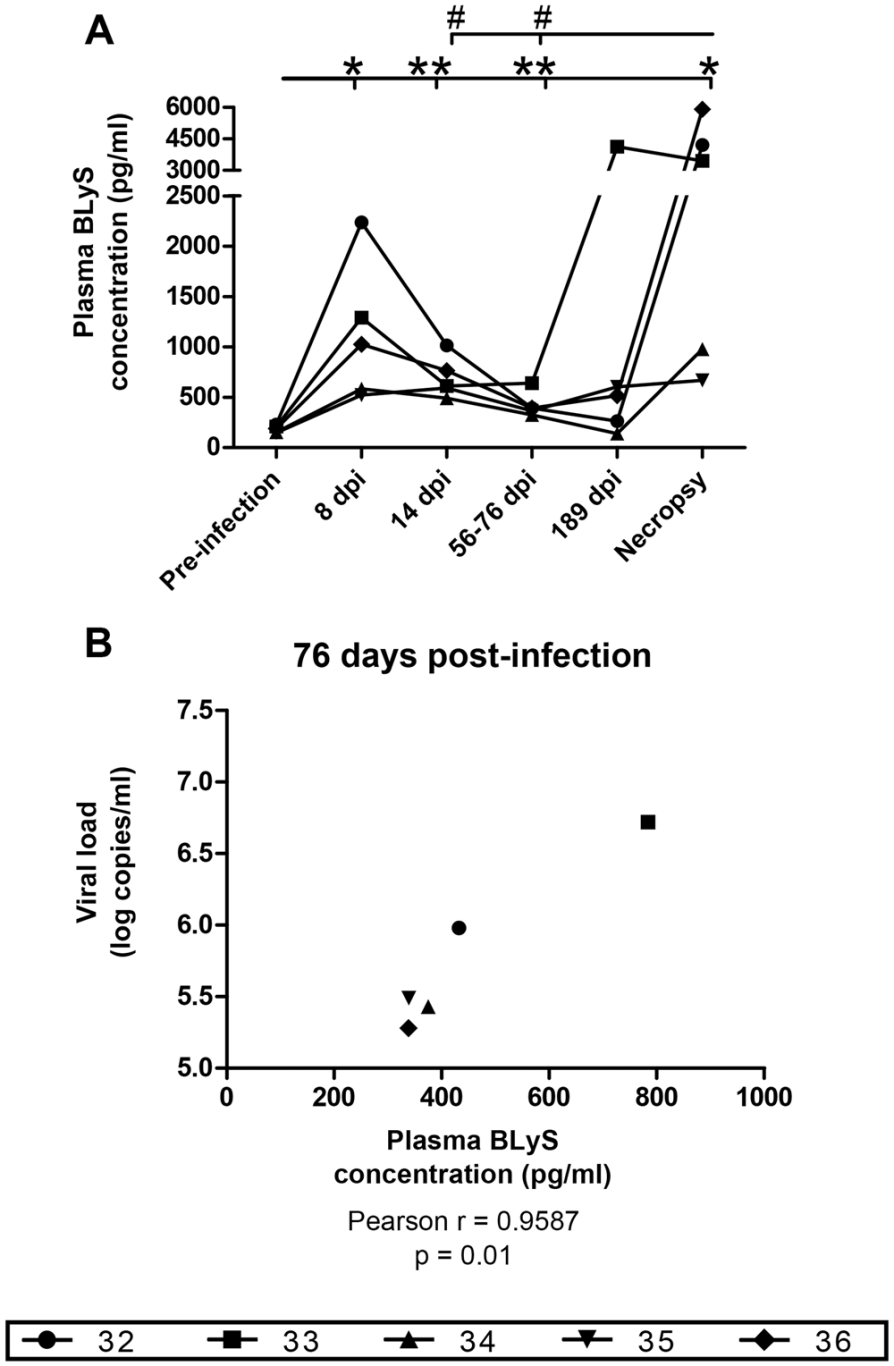
**Longitudinal monitoring of plasma BLyS/BAFF concentrations** (A) Plasma BLyS/BAFF concentrations of 5 SIV-infected macaques were measured by ELISA, and compared to values obtained at pre-infection using the paired Student's *t* test (*) or between time points with repeated-measures ANOVA test followed by Tukey’s post-test (#). Significances levels are shown as *,# p < 0.05, **,## p < 0.001 and ***,###. (B) Pearson correlation between plasma BLyS/BAFF concentrations and viral loads (log copies/ml) after 76 days following SIV infection. dpi, days post infection.

Both the relative percentages of blood mDC sub-populations expressing BLyS/BAFF and levels of BLyS/BAFF expression by these cells increased significantly in the acute phase of infection and remained slightly above pre-infection levels thereafter (Fig 3A and 3B). Except for one animal (#32), the mean percentages and levels of BLyS/BAFF expression by blood monocyte sub-populations remained comparable to pre-infection levels during the course of infection, albeit a significant reduction was observed in the chronic phase of infection (day 182) (Fig 3C and 3D). The mean levels of BLyS/BAFF expression by blood CD4^+^ T-cells increased significantly at three times points (days 8, 56–76 post-infection, and necropsy) (Fig 3E, right panel). Both the mean percentages and levels of BLyS/BAFF expression by blood granulocytes fluctuated considerably throughout infection with significant increases at days 8 and 56–76, and reductions at days 14, 182 and at necropsy (Fig 3F). BLyS/BAFF plasma levels were increased in all macaques, early on and throughout infection (Fig 4A), and the levels of plasma BLyS/BFF correlated with plasma SIV RNA (Fig 4B).

### Longitudinal monitoring of B-cell populations and immunoglobulin concentrations in the blood of SIV-infected macaques

**Fig 5:**
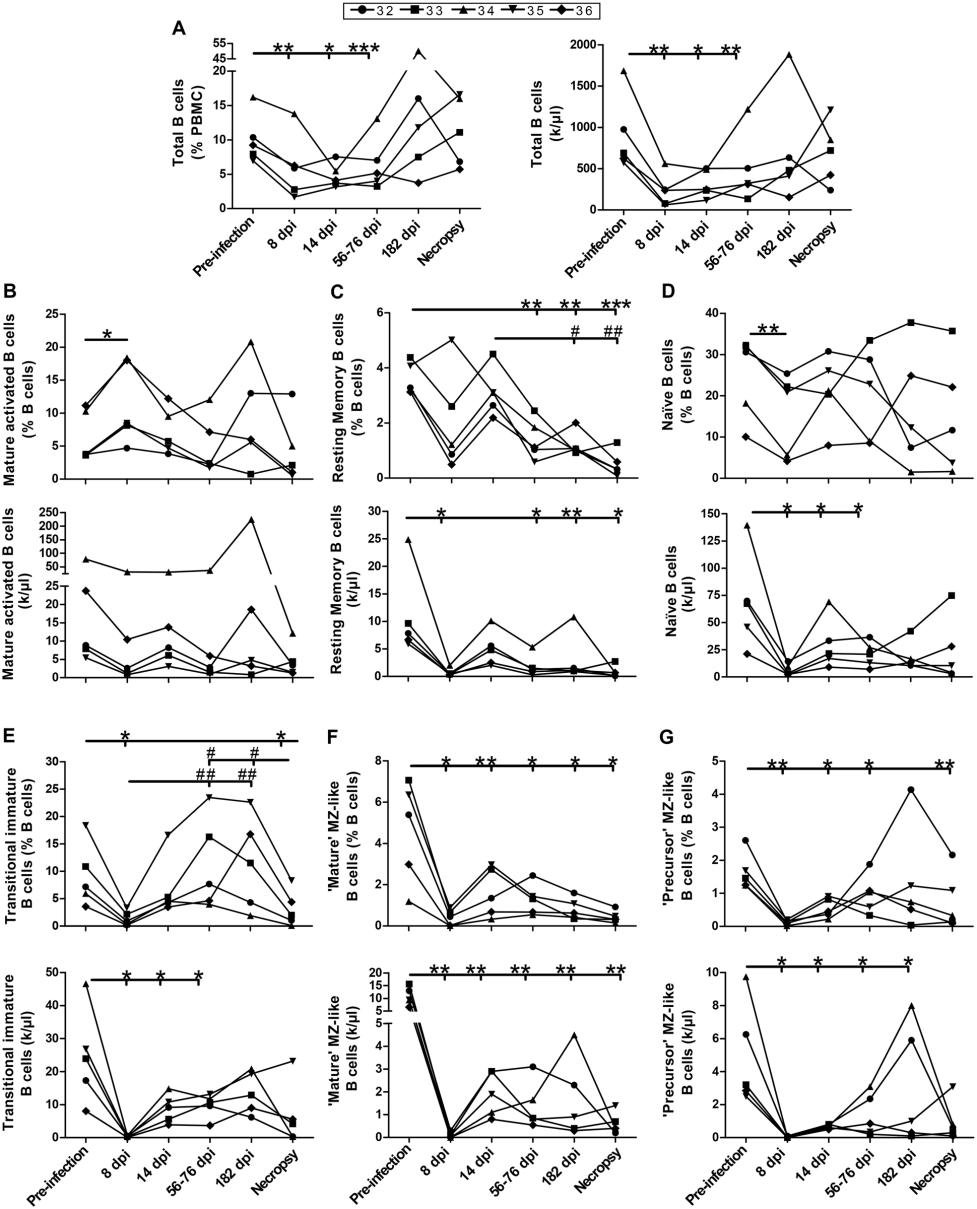
Longitudinal monitoring of B-cell populations. The graphs present the relative frequencies and absolute numbers of (A) total B-cells, (B) mature activated, (C) resting switched memory, (D) naïve, (E) transitional immature (TI), (F) ‘mature’ marginal zone (MZ)-like and (G) ‘precursor’ MZ-like B-cell populations obtained from the blood of 5 SIV-infected rhesus macaques. Values were compared to that obtained at pre-infection stage using the paired Student's *t* test (*) or between time points with repeated-measures ANOVA test followed by Tukey’s post-test (#). Significances levels are shown as *,# p < 0.05, **,## p < 0.001 and ***,###p < 0.0001. dpi, days post-infection.

**Fig 6:**
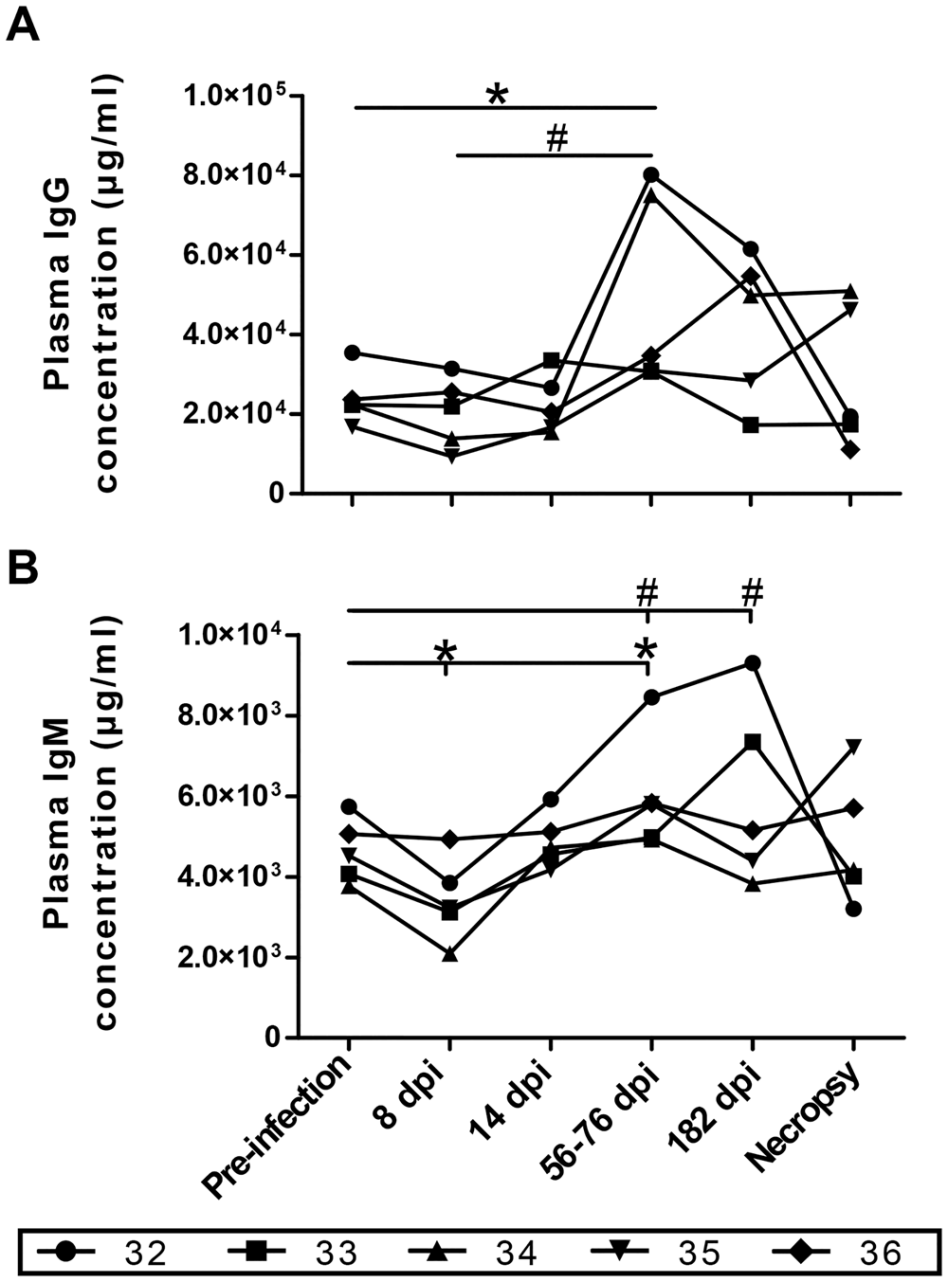
Longitudinal monitoring of plasma IgG and IgM. Plasma (A) IgG and (B) IgM levels of 5 SIV-infected rhesus macaques were measured by ELISA. Concentrations were compared to values obtained at pre-infection stage the paired Student's *t* test (*) or between time points with repeated-measures ANOVA test followed by Tukey’s post-test (#). Significances levels are shown as *,# p < 0.05, **,## p < 0.001 and ***,###p < 0.0001. dpi, days post-infection.

A representative scheme showing the flow-cytometry gating strategy used for the analysis of blood B-cell populations is provided in S2 Fig. Based on the expression of CD20, we have identified in the blood of animals the equivalent of blood B-cell populations which were analyzed in previous human studies [19,20]. Longitudinal analyses of blood CD20^+^ B-cells showed a significant decrease in relative percentages and absolute numbers of total B-cells during the early phase of infection and a trend towards restoration in the chronic phase of infection for most animals (Fig 5A). Although there was a significant increase in the relative frequencies of blood CD20^+^CD27^+^CD21^lo^IgM^-^ mature activated B-cells at day 8 post-infection, globally both frequencies and absolute numbers of mature activated B-cells during the course of infection remained similar to those observed before infection (Fig 5B). The relative percentages and absolute numbers of blood CD20^+^CD27^+^CD21^hi^IgM^-^ switched memory, CD20^+^CD27^-^IgM^+^CD21^hi^CD10^-^ naïve, CD20^+^CD27^-^IgM^+^CD21^+^CD10^+^ TI, CD20^+^CD27^+^IgM^hi^CD21^hi^CD1c^+^ CD10^-^ mature MZ-like and CD20^+^CD27^+^IgM^hi^CD21^lo^CD1c^+^CD10^+^ precursor MZ-like B-cells at all time points following infection were below pre-infection levels albeit we observed a trend towards restoration of these B-cell populations in the chronic phase of infection for most animals (Fig 5C–5G). Both total IgM and IgG plasma concentrations were significantly increased at days 56–76 and 182 post-infection when compared to pre-infection levels (Fig 6).

## Discussion

Similar to what has been previously reported in HIV-1-infected humans [18] and SIV-infected macaques [23,24], we found that blood granulocytes, CD4^+^ T and myeloid cell populations were decreased throughout the course of SIV infection. The relative reduction of these cells in the blood may suggest that they have migrated to peripheral and mucosal sites, as suggested by the increased expression of the tissue tropic CCR7-CCL19 chemokine axis observed in SIV-infected macaques [23]. Importantly, as previously shown in HIV-1-infected humans [19], blood mDCs of SIV-infected macaques expressed high levels of BLyS/BAFF throughout disease progression. In addition, blood CD4^+^ T-cells and granulocytes also expressed high levels of BLyS/BAFF in SIV-infected macaques. Interestingly, the granulocytes were the cell type expressing the highest levels of BLyS/BAFF in the blood of these animals (Fig 2F, right panel). This is a very important novel observation given the recent findings by the group of Cerutti et al; showing that neutrophils are important modulators of first-line MZ B-cells in humans [25]. As such, the MZ B-cell pool is highly influenced by BLyS/BAFF [13,14] and we have previously reported that frequencies of MZ-like B-cells presenting increased IL-10 expression profiles are increased in the blood of HIV-1-infected individuals [19,20]. Although, MZ-like B-cells were not increased in the blood of SIV-infected macaques (Fig 5F and 5G), cells with similar phenotypes and dysregulated activation profiles have been shown to be reduced in blood and to accumulate in mucosal tissues of infected animals [26–29].

Thus, similar to that observed in human HIV-1 infection [19,20], the increased BLyS/BAFF expression levels found in SIV-infected macaques are concomitant with a dysregulated blood B-cell compartment. Total B-cells were decreased significantly during the early phase of SIV infection and remained below pre-infection levels throughout the course of infection for most animals (Fig 5A). This is in agreement with the demonstration that SIV has been shown to rapidly induce B-cells to accumulate in periphery and intestinal mucosa following infection [30]. As described for chronically HIV-infected individuals [19,20,31], resting switched memory B-cells were diminished in the blood of SIV-infected macaques. This finding is consistent with altered recall memory responses [32] and likely reflects perturbed germinal center reactions observed in lymphoid organs of SIV-infected macaques [27], and which have also been reported in HIV-infected humans [33] and HIV-Tg mice [12]. High BLyS/BAFF levels may significantly influence the ability to mount efficient B-cell responses and disease progression, favouring dysregulated populations and polyclonal expansion at the expense of protective refined high affinity responses. As such, polyclonal activation was reflected in the blood of SIV-infected macaques by IgM and IgG hyperglobulinemia and by a significant increase in the relative frequencies of blood mature activated B-cells and of B-cells presenting a CD27^-^CD21^-/lo^ exhausted phenotype (S3 Fig) [31]. Exhausted populations and serum hyperglobulinemia reported here are consistent with similar findings obtained in the context of SIV [27,30].

As we have previously shown for HIV-1-infected progressors [19], BLyS/BAFF plasma levels were also increased in SIV-infected animals, as soon as day 8 post-infection and throughout the course of infection (Fig 4A), and correlated with viral loads (Fig 4B). This is in agreement with the findings by Chaoul et al [30], who also observed increased BLyS/BAFF in the plasma and intestinal mucosa of SIV-infected macaques. The increased expression of BLyS/BAFF in SIV-infected macaques is likely to result from direct and indirect effects of the virus [34]. Possible explanations as to why BLyS/BAFF levels fluctuated during the course of infection may involve the time of sampling which corresponds to different phases of disease progression towards AIDS, which are likely to influence immune status. Interestingly, animal #32 presented a better control of viremia when compared to the other animals, and to a certain extent animal #34 also presented lower viral loads following the acute phase. As found for our human studies, there are intrinsic variations in the overall immune context and management of disease progression, which are likely to influence levels of BLyS/BAFF [19], and animal #32 as well as animal #34 are representative of this reality. In fact, as shown in Fig 2, we found high levels of BLyS/BAFF on blood populations of these animals, which were concomitant with B-cell dysregulations. However, these animals presented a slower progression to AIDS as they survived for a longer period when compared to the other animals (S1 Table). Interestingly, we have recently described that the viral factor Nef, found to be released and detected in the blood of HIV-1-infected individuals [35–38], is involved in driving BLyS/BAFF over-expression in human mDCs, even following successful control of viremia suggesting a reservoir source [38]. It remains however to be established as to whether SIV Nef can modulate BLyS/BAFF expression in macaques. Although Nef may be directly involved in BLyS/BAFF over-expression, the overall inflammatory condition and elements of microbial translocation reported in the context of HIV and SIV, such as LPS, [34] may also likely influence BLyS/BAFF expression, as suggested by our recent observations [38].

Similar to the HIV/SIV context, BLyS/BAFF is increased in several autoimmune disorders [7,39,40], and recent therapeutic trials using anti-BLyS/BAFF (Benlysta/Belimumab) in patients with systemic lupus erythematosus (SLE) have been successful in inducing remission [41]. Furthermore, growing evidence suggest blocking BLyS/BAFF will have important implications for cancer, allergy, and inflammatory diseases [42]. Increased BLyS/BAFF levels have also been reported in the context of B-cell depletion using anti-CD20 (Rituximab) treatment of human patients presenting B-cell lymphoma [43]. Following treatment, serum levels of BLyS/BAFF and B-cell numbers eventually reciprocally stabilize. Suggesting that the binding of BLyS/BAFF to its receptors expressed on B-cells is involved in regulating soluble BLyS/BAFF levels [43]. In such circumstances, it is likely that the rise in serum BLyS/BAFF levels is related to both under-consumption as well as over-production. Indeed, it has been recently shown that anti-CD20, which mechanism of action involves NK cells and ADCC effector functions, also promotes BLyS/BAFF production by NK cells [44]. As such, neutralization of BLyS/BAFF with Belimumab restored sensitivity of BLyS/BAFF prone chronic lymphoid leukemia cells to Rituximab, supporting there is a benefit in combining anti-CD20 and anti-BLyS/BAFF in certain circumstances [44]. In the context of chronic infection and inflammation such as encountered with HIV/SIV, we find increased BLyS/BAFF levels are related to increased production mediated by factors such as Nef and LPS [38], and may possibly also involve under/deregulated-consumption as percentages of total B-cells are decreased in blood. Albeit it is likely that B-cells accumulate in peripheral sites, as reported in the context of SIV [30] and as we reported in the HIV-Tg mouse model [12]. Furthermore, we find increased relative frequencies of precursor MZ-like B-cells in the blood,of HIV-1-infected progressors [19,20] and BLyS/BAFF promotes TACI expression on this population *in vitro* (JCC, JP unpublished). Although Rituximab is used for treatment of B-cell lymphomas in HIV-infected patients, it is controversial and associated with high risk of infections in patients with blood CD4^+^ T-cell counts <100 cells/mm^3^ [45,46]. It is likely that in such circumstances with HIV/SIV, BLyS/BAFF levels are not homeostatically restored following Rituximab treatment and continue to be fuelled by unresolved chronic stimulation, and therefore contribute to sustained B-cell dysregulations. This strengthens the point that in such situations, blocking excess BLyS/BAFF is perhaps needed in order to restore B-cell homeostasis. Further studies are thus mandatory in this field.

## Conclusions

The present study in SIV-infected macaques and previous studies in HIV-1-infected humans [19,20,38], provide strong evidence that BLyS/BAFF is a key regulator of SIV/HIV-induced immune dysregulation and disease progression, that is highly-conserved among primates. Moreover, these findings validate the SIV-rhesus macaque model for future pre-clinical studies to use anti-BLyS/BAFF to test if we can restore immune homeostasis and effectiveness by reducing pro-inflammatory conditions and viral reservoirs.

## Supporting Information

S1 Fig Representative gating strategy for flow-cytometry analysis of macaque blood myeloid dendritic cells (mDC), monocytes, CD4^+^ T-cells and granulocytes. Representative plot showing gating strategy on live blood PBMCs of 5 SIV-infected rhesus macaques. Granulocytes are characterized with a high SSC/FSC profile. Cells presenting a lower SSC profile were discriminated upon their expression of either HLA-DR or CD3, for T lymphocytes, the latter selected for CD4 expression. Cells expressing HLA-DR were selected by exclusion of CD20 and CD14, and these mDCs were characterized upon their CD16 and CD1c expression. Monocytes were selected with a higher FSC profile, determined as CD3^-^CD20^-^HLA-DR^+^, and characterized upon their CD16 and CD14 expression.
(TIF)

S2 Fig Representative gating strategy for flow-cytometry analysis of macaque blood B-cell populations. Representative plot showing gating strategy on live blood PBMCs of 5 SIV-infected rhesus macaques. Total CD20^+^ B-cells were selected based on expression of CD27 and/or IgM, and levels of CD21. CD1c and CD10 expression were used for further characterisation of blood MZ and TI B-cell populations respectively, as reported [20]. Quadrants were set based on the expression values obtained with fluorescence minus one (FMO) and isotype controls. Mature activated B-cells are defined as CD20^+^CD27^+^IgM^-^CD21^lo^CD1c^-^CD10^-^, resting switched memory B-cells are CD20^+^CD27^+^IgM^-^CD21^hi^CD10^-^, precursor marginal-zone (MZ)-like B-cells are CD20^+^CD27^+^IgM^+^ CD21^lo^CD1c^+^CD10^+^, mature MZ-like B-cells are CD19^+^CD27^+^IgM^+^CD21^hi^CD1c^+^CD10^-^ and transitional immature (TI) B-cells are CD20^+^CD27^-^IgM^+^CD21^hi^CD1c^-^CD10^+^.
(TIF)

S3 Fig Longitudinal Analysis of B-cell populations According to CD27 and CD21 expression profiles. The graphs present the relative frequencies of CD20^+^ B-cells expressing (A) CD27^+^CD21^hi^,which include resting memory and mature marginal zone (MZ) populations (B) CD27^+^CD21^lo^,which include mature activated and precursor MZ populations (C) CD27^-^CD21^hi^, which include naïve resting and transitional immature (TI) populations and finally (D) CD27^-^CD21^-/lo^ which include tissue memory like exhausted B-cells B-cells were obtained from the blood of 5 SIV-infected rhesus macaques. dpi, days post-infection.
(TIF)

S4 Fig Flow-Cytometry Control for BLyS/BAFF expression. (TIF)

S1 Table Characteristics of the SIV-infected Rhesus Macaques used in this study. (TIF)
