# Supplementary figures and images for: High Expression Levels of BLyS/BAFF by Blood Dendritic Cells and Granulocytes Are Associated with B-cell dysregulation in SIV-Infected Rhesus Macaques
Source: PLoS One. 2015 Jun 24;10(6):e0131513. doi: 10.1371/journal.pone.0131513 (PMC4479440; doi:10.1371/journal.pone.0131513)

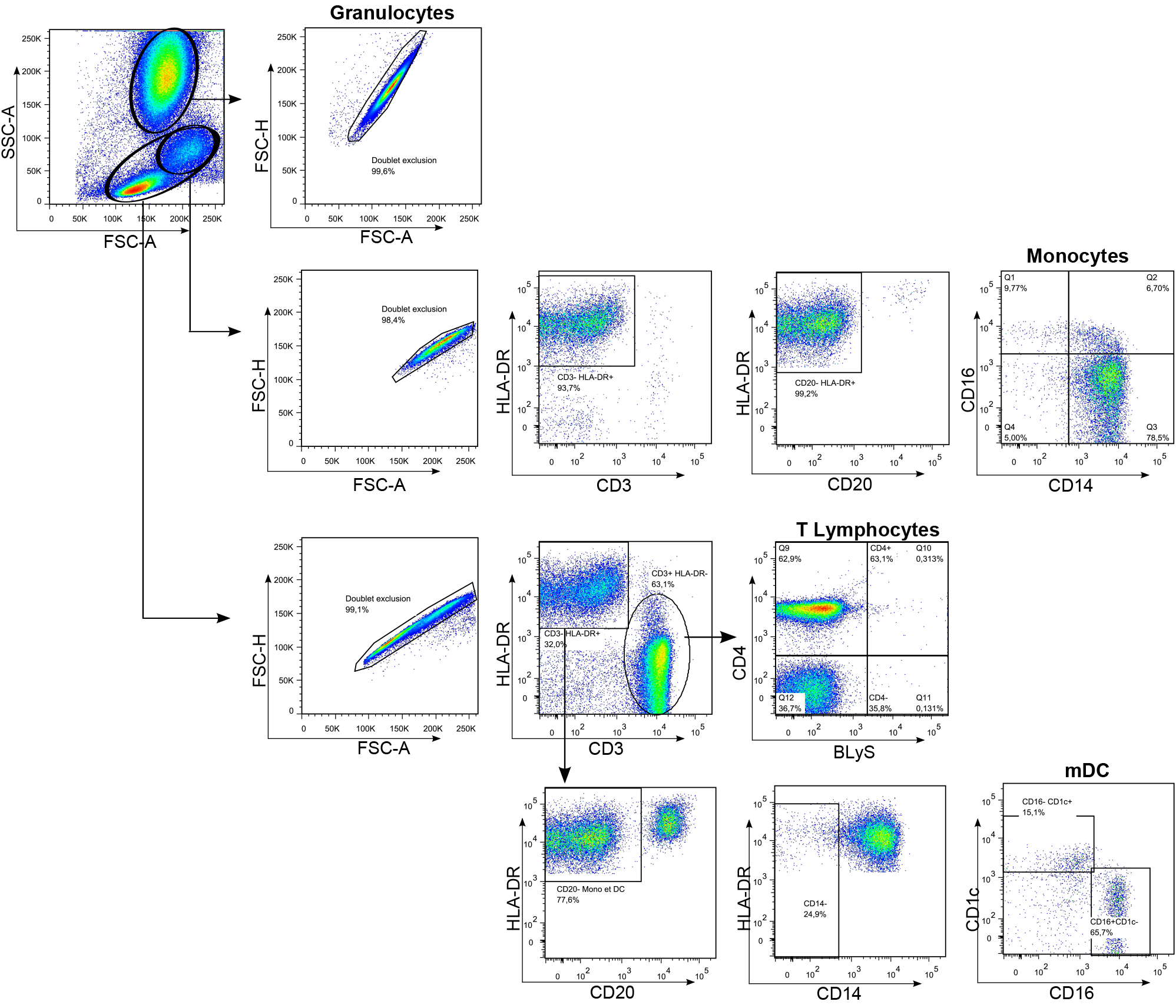

Supplement: S1 Fig — Representative plot showing gating strategy on live blood PBMCs of 5 SIV-infected rhesus macaques. Granulocytes are characterized with a high SSC/FSC profile. Cells presenting a lower SSC profile were discriminated upon their expression of either HLA-DR or CD3, for T lymphocytes, the latter selected for CD4 expression. Cells expressing HLA-DR were selected by exclusion of CD20 and CD14, and these mDCs were characterized upon their CD16 and CD1c expression. Monocytes were selected with a higher FSC profile, determined as CD3-CD20-HLA-DR+, and characterized upon their CD16 and CD14 expression. (TIF) [file pone.0131513.s001.tif]

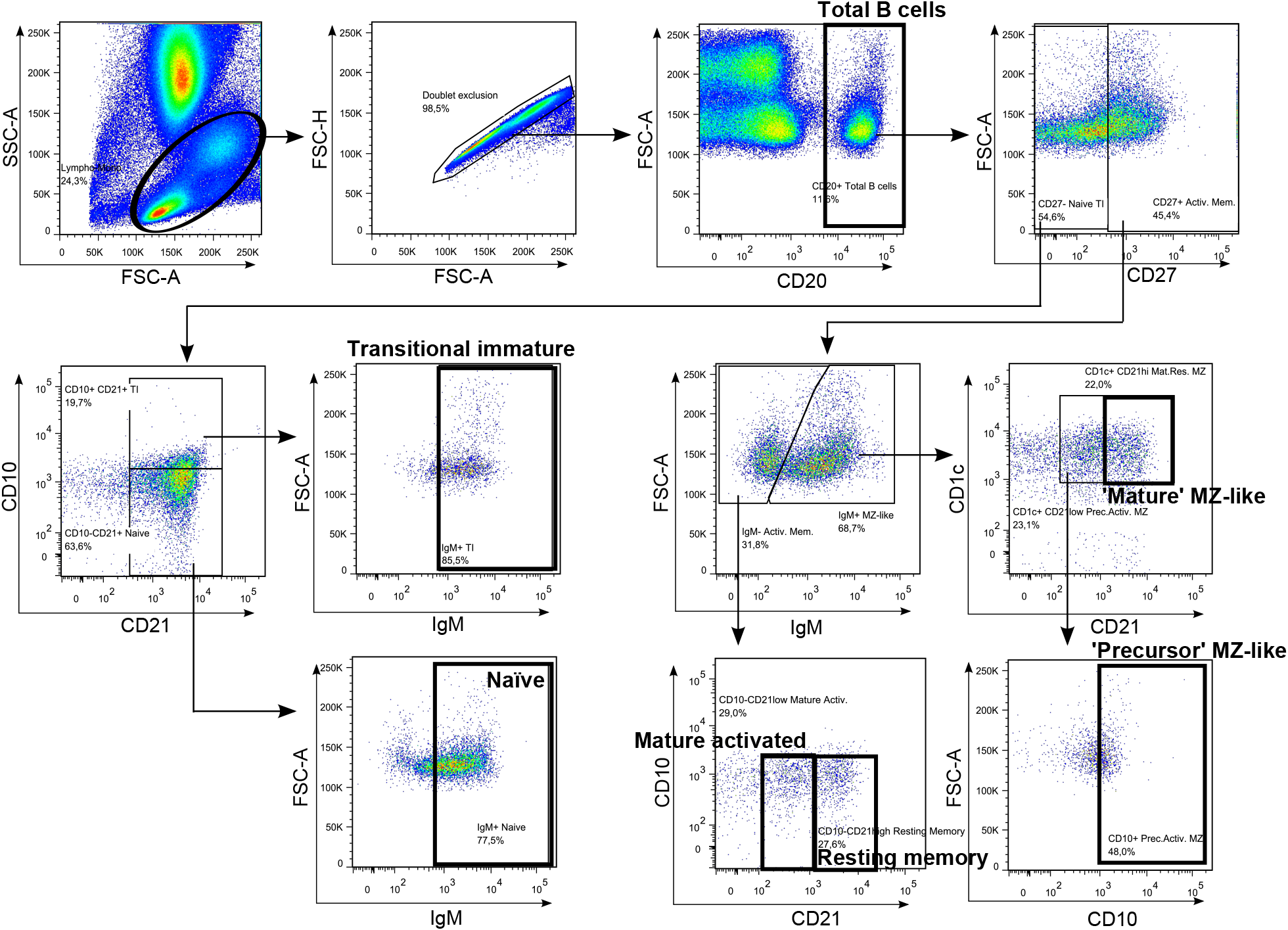

Supplement: S2 Fig — Representative plot showing gating strategy on live blood PBMCs of 5 SIV-infected rhesus macaques. Total CD20+ B-cells were selected based on expression of CD27 and/or IgM, and levels of CD21. CD1c and CD10 expression were used for further characterisation of blood MZ and TI B-cell populations respectively, as reported [20]. Quadrants were set based on the expression values obtained with fluorescence minus one (FMO) and isotype controls. Mature activated B-cells are defined as CD20+CD27+IgM-CD21loCD1c-CD10-, resting switched memory B-cells are CD20+CD27+IgM-CD21hiCD10-, precursor marginal-zone (MZ)-like B-cells are CD20+CD27+IgM+ CD21loCD1c+CD10+, mature MZ-like B-cells are CD19+CD27+IgM+CD21hiCD1c+CD10- and transitional immature (TI) B-cells are CD20+CD27-IgM+CD21hiCD1c-CD10+. (TIF) [file pone.0131513.s002.tif]

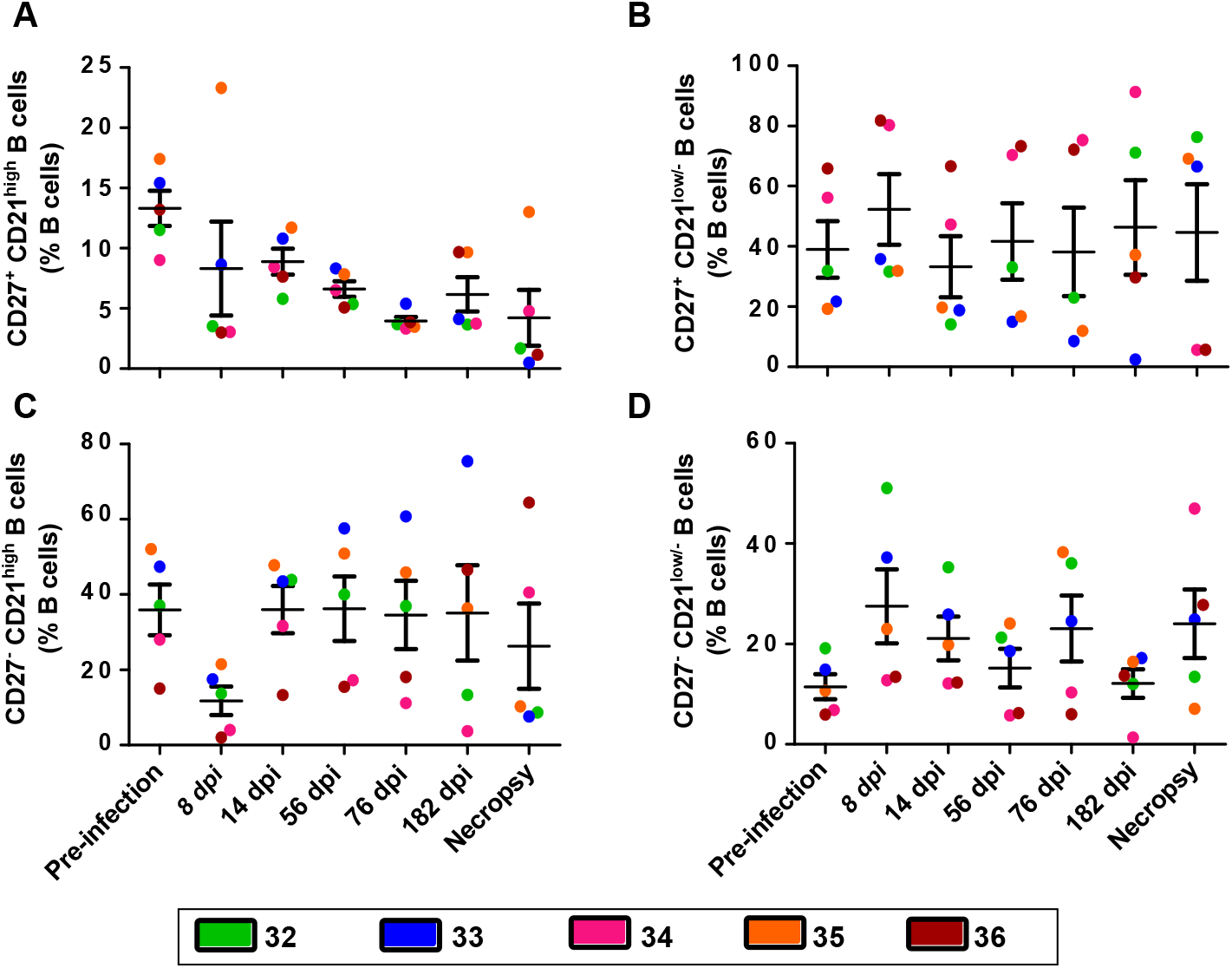

Supplement: S3 Fig — The graphs present the relative frequencies of CD20+ B-cells expressing (A) CD27+CD21hi,which include resting memory and mature marginal zone (MZ) populations (B) CD27+CD21lo,which include mature activated and precursor MZ populations (C) CD27-CD21hi, which include naïve resting and transitional immature (TI) populations and finally (D) CD27-CD21-/lo which include tissue memory like exhausted B-cells B-cells were obtained from the blood of 5 SIV-infected rhesus macaques. dpi, days post-infection. (TIF) [file pone.0131513.s003.tif]

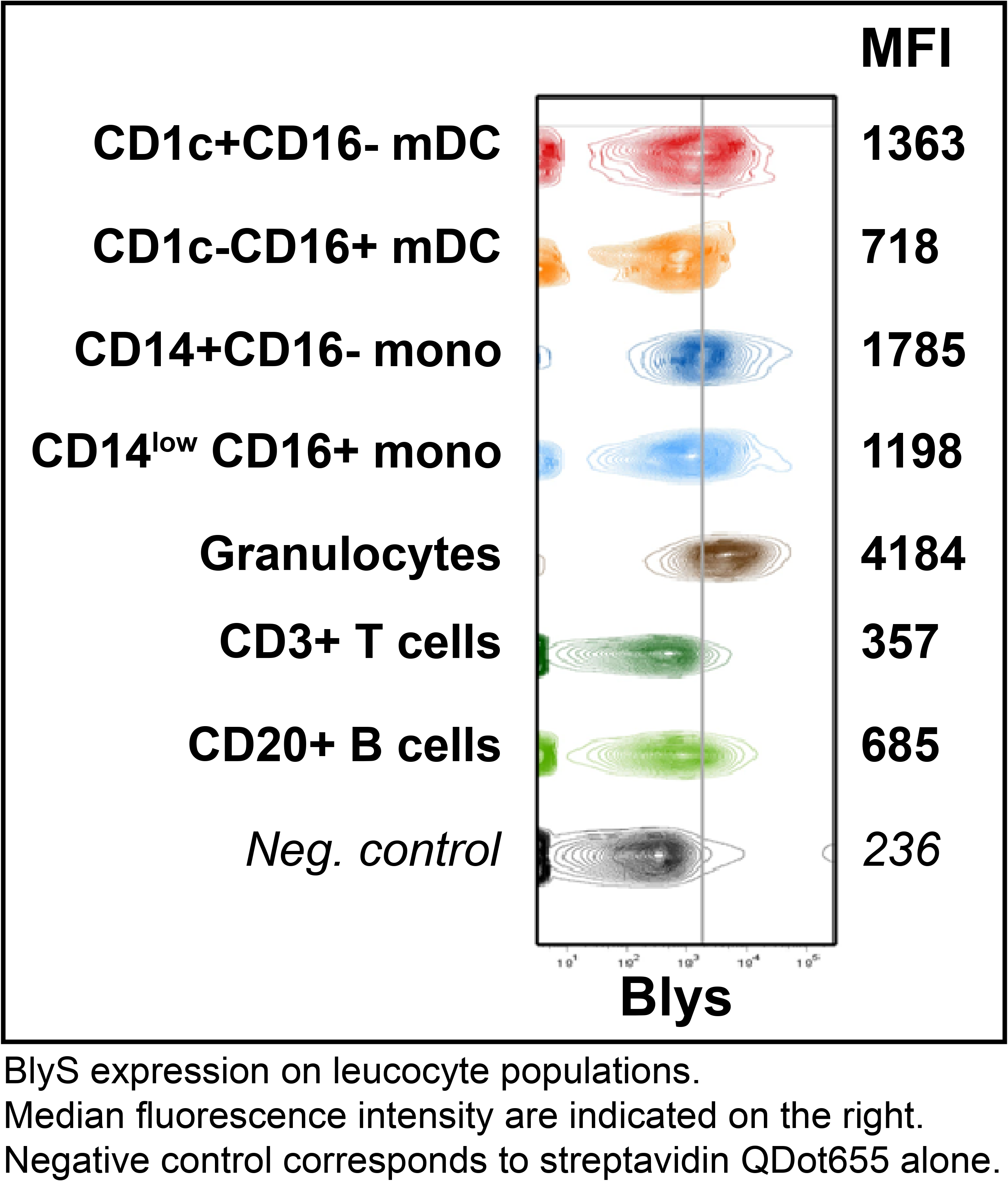

Supplement: S4 Fig — (TIF) [file pone.0131513.s004.tif]

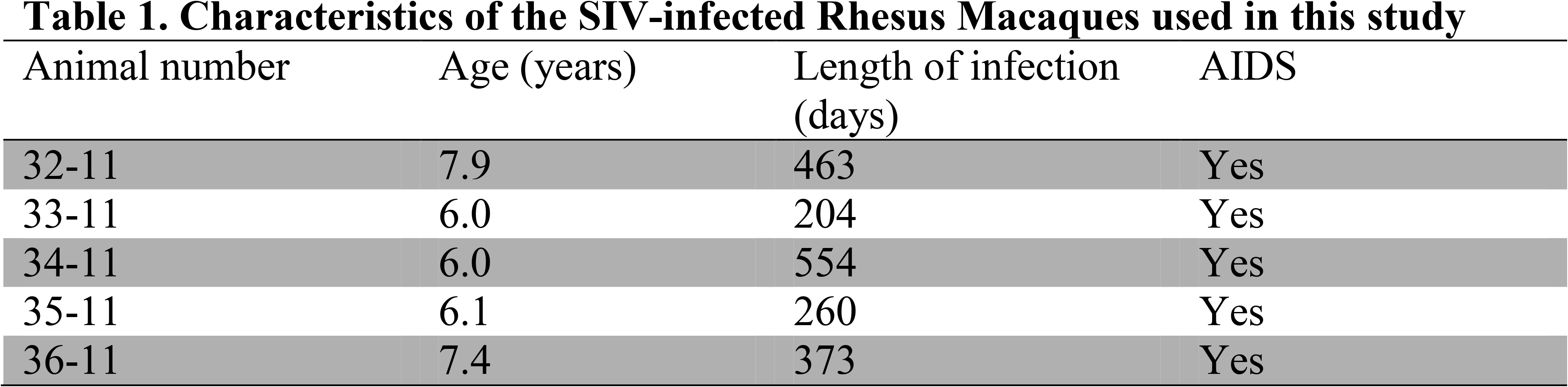

Supplement: S1 Table — (TIF) [file pone.0131513.s005.tif]
